# Facilitating Collaboration in Rare Genetic Disorders Through Effective Matchmaking in DECIPHER

**DOI:** 10.1002/humu.22842

**Published:** 2015-08-20

**Authors:** Eleni A. Chatzimichali, Simon Brent, Benjamin Hutton, Daniel Perrett, Caroline F. Wright, Andrew P. Bevan, Matthew E. Hurles, Helen V. Firth, Ganesh J. Swaminathan

**Affiliations:** ^1^ Wellcome Trust Sanger InstituteWellcome Trust Genome CampusHinxtonCambridgeCB10 1SDUnited Kingdom; ^2^ Cambridge University Department of Medical GeneticsAddenbrooke's HospitalCambridgeCB2 2QQUnited Kingdom

**Keywords:** genetic disorders, rare diseases, genotype‐phenotype correlation, MatchMaker Exchange

## Abstract

DECIPHER (https://decipher.sanger.ac.uk) is a web‐based platform for secure deposition, analysis, and sharing of plausibly pathogenic genomic variants from well‐phenotyped patients suffering from genetic disorders. DECIPHER aids clinical interpretation of these rare sequence and copy‐number variants by providing tools for variant analysis and identification of other patients exhibiting similar genotype–phenotype characteristics. DECIPHER also provides mechanisms to encourage collaboration among a global community of clinical centers and researchers, as well as exchange of information between clinicians and researchers within a consortium, to accelerate discovery and diagnosis. DECIPHER has contributed to matchmaking efforts by enabling the global clinical genetics community to identify many previously undiagnosed syndromes and new disease genes, and has facilitated the publication of over 700 peer‐reviewed scientific publications since 2004. At the time of writing, DECIPHER contains anonymized data from ∼250 registered centers on more than 51,500 patients (∼18000 patients with consent for data sharing and ∼25000 anonymized records shared privately). In this paper, we describe salient features of the platform, with special emphasis on the tools and processes that aid interpretation, sharing, and effective matchmaking with other data held in the database and that make DECIPHER an invaluable clinical and research resource.

## Introduction

Genetic diseases arise from errors in an individual's genome, which disrupt the normal functions of genes. These errors range from small changes in one or more critical genes to structural changes such as duplications, deletions, inversions, and translocations of larger sections of the genome affecting one or more genes. In the absence of routine genetic testing, initial clues to an underlying genetic disorder are given by the external manifestation (phenotype) of patients referred to the clinic. While some phenotypes are highly suggestive of an underlying genetic disorder (e.g., Progeria [Eriksson et al., 2003] or Downs syndrome [Lejeune et al., 1959]), genetic testing is necessary to confirm a molecular diagnosis. The relationship between phenotypes and underlying genetic causes (genotypes), however, is far from straightforward due to both phenotypic and genetic heterogeneity. The rapid advances and falling costs of genomic technologies have accelerated the routine use of high‐resolution genetic methods such as array‐comparative genomic hybridization, single‐nucleotide polymorphism (SNP) genotyping and exome sequencing in a clinical setting [Shaw‐Smith et al., 2004; Stankiewicz and Beaudet, 2007] as well as in research. This has resulted in major advances in the ability to diagnose genetic diseases and has led to identification of many new causative genes [Ng et al., 2010; Cooper et al., 2011; Deciphering Developmental Disorders Study, 2015].

There are now over 7,000 recognized rare diseases, identified as those diseases that occur at very low rates among the population (five in 10,000 [EU], or less than 200,000 people [USA]), most with an underlying and as‐yet unidentified genetic cause (source: http://www.eurordis.org/). The rarity and novelty of the causative genetic factors means that determining whether a particular genetic variant is pathogenic or benign and its correlation with respect to a patient's disease phenotype is challenging when viewed in isolation. While the identification of another patient with similar aetiology helps in narrowing causative factors, a large‐scale collation and comparison of phenotypes in patients with similar genomic changes provides greater certainty that an observed variant is causal for the phenotype. Such comparative studies also assist in the identification of new syndromes and highlight genes that may be causal in a particular disorder.

DECIPHER (https://decipher.sanger.ac.uk) [Firth et al., 2009; Swaminathan et al., 2012; Bragin et al., 2014] was established in 2004 to initiate an international collaborative matchmaking effort to aid diagnosis by finding patients with shared genetic and phenotypic features. DECIPHER accomplishes this by providing a web‐based platform that permits:

1. Secure deposition of plausibly causative genetic findings with associated phenotypes
2. Visualization of deposited information in a genome‐wide context, with annotation of recognized functional elements
3. Delineation of genes based on their association with disease, or their implication in developmental disorders, or their likelihood to be haploinsufficient [Huang et al., 2010]
4. Identification of other deposited patients with a genetic (positional or functional) overlap and similar phenotypes
5. Contact mechanisms to encourage collaboration and information exchange between users regarding patients of interest.

**Figure 1:**
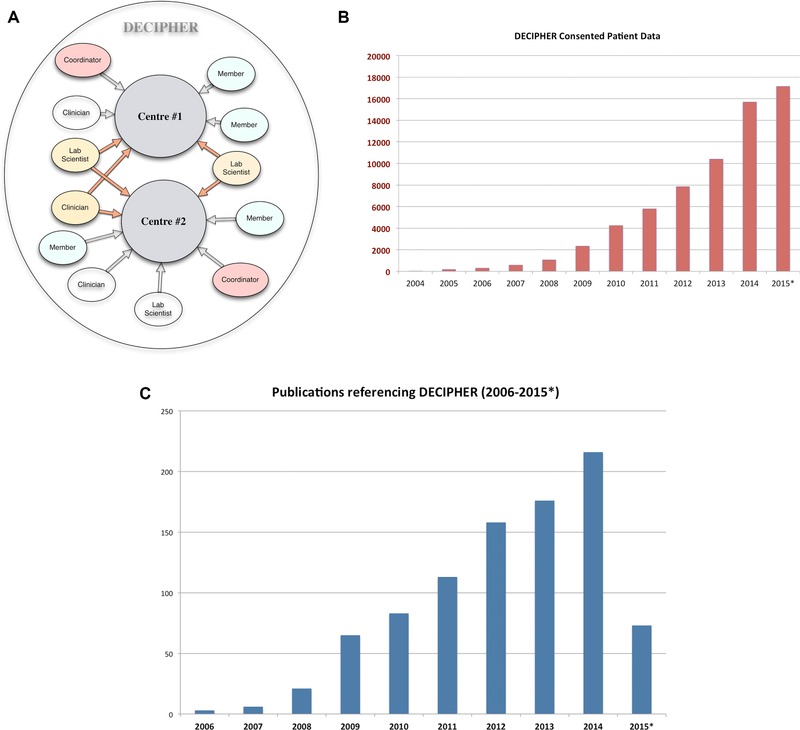
**A**: The DECIPHER community consists of centers of clinical genetics and associated research groups. Each center has a designated clinical coordinator and authorized users with distinct roles and access permissions that allow them to access, edit, and/or deposit data. **B**: The DECIPHER database contains over 17,000 anonymized patient‐consented records (genotype and phenotype) available to all users for matchmaking, contact, or collaboration. **C**: A metric of the success of collaboration is the increased number of peer‐reviewed publications that have used data from DECIPHER for the identification of previously unidentified syndromes and/or novel genes implicated in genetic disorders.

To our knowledge, DECIPHER was the first resource of its kind committed to encouraging genomic matchmaking through information displayed and derived on its website and by facilitating contact and collaboration between users. Over 700 peer‐reviewed publications have resulted from the use of DECIPHER through user‐to‐user collaborations and by the use of data available from our website since its inception (Fig. 1C). DECIPHER has also assisted in the identification of new syndromes [Shaw‐Smith et al., 2006; Zahir et al., 2007; Engels et al., 2009; Malan et al., 2009; Barge‐Schaapveld et al., 2011; Molin et al., 2012; Thevenon et al., 2012]. At the time of writing, DECIPHER contains genotype‐linked phenotype data for over 51,500 patients deposited from academic clinical genetics centers from 40 countries, of which 17,000+ are consented for anonymous public sharing, and another 18,000 are shared privately between consortia (groups of depositing centers) established within the DECIPHER framework (Fig. 1B). DECIPHER provides a secure web interface for users to deposit their patient phenotype and variant data, and to carry out comparative analysis of their patient data against all the data that is available to them (see *Data Sharing* below). With informed patient consent, linked‐anonymized variant and phenotype data are made available to all users via the DECIPHER website, selected genome browsers such as Ensembl and UCSC Genome Browser [Cunningham et al., 2015; Rosenbloom et al., 2015] or as a bulk dataset for bioinformatics research. In addition to powerful search functionality, DECIPHER provides users with a suite of integrated analysis and visualization tools designed to aid in the interpretation of deposited sequence or copy‐number variants (CNVs). In this article, we describe the DECIPHER database and web service, and highlight the features that make it an invaluable resource for genomic medicine and genetic research, with an emphasis on tools and methods that enable effective matchmaking.

## The DECIPHER Community

DECIPHER engages a community of depositing centers that includes academic hospitals and departments of clinical genetics, their related genetics faculties, and research groups that study the genetics of rare diseases. Each constituent member site is assigned a project identifier and is in turn made up of users with specific, designated roles and access rights that govern their interaction with the DECIPHER platform (Fig. 1A). User roles include clinicians (those responsible for the patient data in DECIPHER), laboratory scientists, and members (nurses, junior doctors, and genetic counselors). Each project also has a coordinator role, fulfilled by a senior clinician in the project, for approving membership requests from other users wishing to join the project and for its overall governance. Authorized users of DECIPHER may belong to one or more projects, or groups of projects termed “consortia.” Such consortia expand beyond the boundaries of geographical locations and across different specialties, and enable access to exclusive patient data that may not have been consented for public sharing. Some examples of consortia with multiple linked projects within the DECIPHER framework include the Deciphering Developmental Disorders Study (DDD) [Wright et al., 2014], eye disorders, genetics of dermatology, and a pilot UK National Health Service (NHS) consortium. Membership of DECIPHER is provided solely for the purposes of facilitating improved diagnosis through deposition, analysis, and subsequent sharing of patient phenotype‐linked variant data following informed patient consent. The deposition process can be conducted asynchronously either in isolation or as a bulk upload.

## Data Deposition and Sharing

DECIPHER offers a secure, password‐protected, online interface and confidential data repository that enables the deposition of genetic data with associated phenotypes and some patient metadata (age, gestation in weeks for prenatal data, sex, affected status of parents, photographs, internal hospital reference, notes) from patients with rare disorders. Some deposited information is only available to the depositing project (hospital reference number, notes) and any consortium the project may be part of (photographs for example). Where consent from the patient is already available, this can be indicated at deposition, thereby making anonymized data from the patient immediately available publicly. Every patient in DECIPHER is assigned a unique identifier that, if known by a clinician or patient who has access to this record, may be used to access the patient pages directly for those with access to this record (e.g., https://decipher.sanger.ac.uk/patient/283351 for DECIPHER patient 283351). The information in the patient pages are distributed between tabs, providing details about the patient, genetic variant(s), phenotype(s), and so on. Following deposition, DECIPHER aids matchmaking by presenting information about other accessible patients in the database that overlap the variant(s) of the patient and by highlighting any shared phenotypes (see *Finding Similar Patients* below).

### Genetic Data

Anonymized genetic data in DECIPHER may include CNVs as well as sequence variants including SNVs (single‐nucleotide variants) and InDels. Additional information about the variant, pathogenicity, inheritance and contribution to phenotype, may also be provided. DECIPHER also supports the deposition of variants from the mitochondrial genome. The deposition interface validates the information provided to ensure that it is internally consistent (e.g., chromosomal location) and tallies with genomic reference data (based on the transcript identifier and reference alleles).

Interpretation of deposited genetic data takes place in real time to permit further analysis and visualization of variants in a genomic context. DECIPHER offers a suite of integrated analysis tools designed to aid in the discrimination and identification of pathogenic sequence variations. For CNVs, this includes a list of genes affected by the variant with properties including their propensity to be haploinsufficient [Huang et al., 2010], association with developmental disorders (DDG2P) [Wright et al., 2014], and Mendelian diseases (OMIM and OMIM Morbid) [Amberger et al., 2015]. In addition, for deposited SNVs and InDels, consequence predictions from the Ensembl variant effect predictor (VEP) [McLaren et al., 2010] are calculated and displayed. This information contains predicted consequences using sequence ontology [Eilbeck et al., 2005] terms, as well as missense pathogenicity scores PolyPhen [Adzhubei et al., 2010] and SIFT [Kumar et al., 2009] predictions for all known transcripts with associated HGVS [den Dunnen and Antonarakis, 2000] identifiers. To facilitate quick interpretation, this section also contains a graphical breakdown (in the form of a pie chart) of the Ensembl VEP annotation.

### Phenotype Data

**Figure 2:**
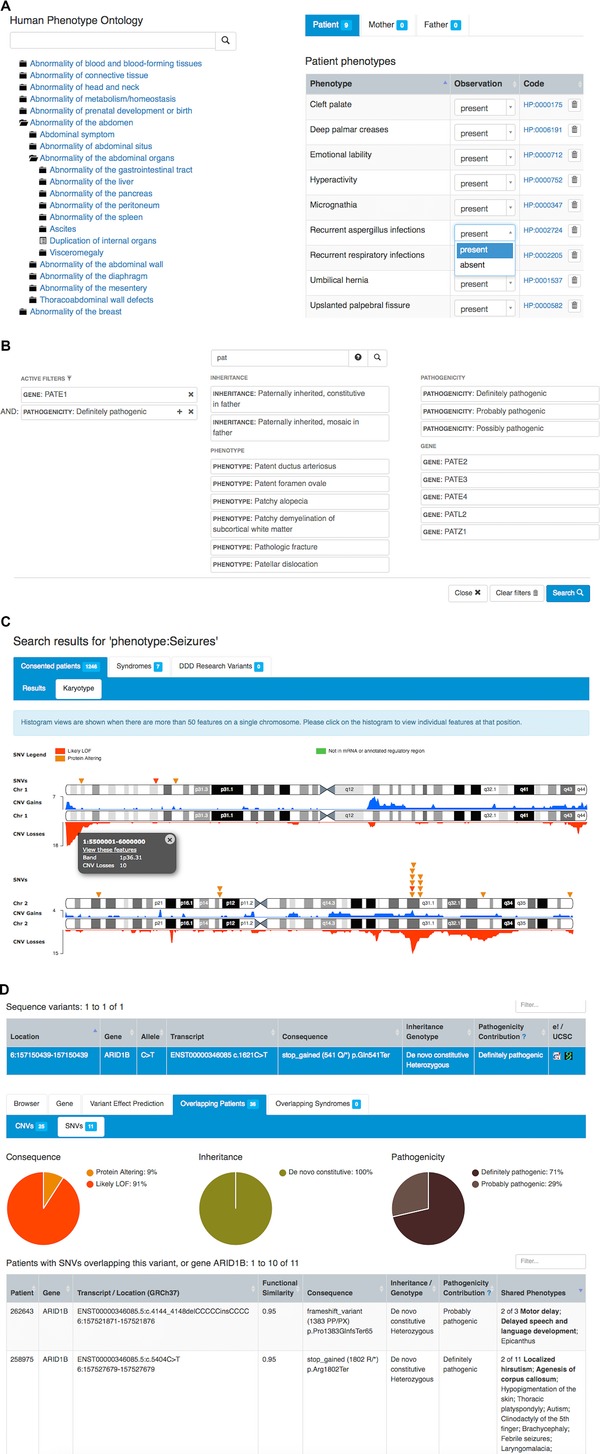
DECIPHER data entry and analysis interface. **A**: Phenotype entry in DECIPHER is based on the Human Phenotype Ontology (HPO) tree and provides a simple drag‐and‐drop interface to quickly record observed or absent patient and parental phenotypes. **B**: DECIPHER search allows querying the database using a combination of terms and is aided by an autosuggest facility to quickly find term of interest. **C**: An interactive histogram representation of the karyotype for a search on phenotype “seizures” showing a peak in the region of 2q24.3 encompassing the *SCN2A* gene. Individual inverted triangles represent sequence variants, whereas copy‐number variants are shown in red (deletion) and blue (duplications). **D**: An *ARID1B* gene sequence variant in DECIPHER and other records in the database overlapping the same gene. DECIPHER assists the identification of other patients in the database that overlap a position using a functional overlap score and highlights shared phenotypes in these patients. Pie charts provide a visual aid to the breakdown of the positional overlap based on various criteria.

Recording the observed clinical phenotype constitutes a cornerstone of the DECIPHER project and is a principal component of matchmaking. Structured phenotypes based on the human phenotype ontology (HPO) (Robinson and Mundlos, 2010), a comprehensive ontology used for the standardization of phenotypic terms in humans, provide a way of quickly evaluating relationships between patients with similar disorders. In addition to the presence of a phenotype in a patient, specific absences of an expected phenotype may also be recorded. Phenotype information for both the patient and the parents can be recorded to aid the interpretation of inheritance of phenotypes and associated genotypes. DECIPHER offers a phenotyping tool that combines searching the HPO tree with “drag‐and‐drop” functionality to quickly add relevant phenotypes to the patient record. (Fig. 2A).

### Data Sharing

There are currently two distinct categories of data sharing within DECIPHER. First, at the patient level, anonymized data may become publicly available after the patient has granted explicit consent. Second, DECIPHER offers granularity of sharing at the variant level. The depositors can single out one or more variants in a patient, and independently change their sharing status to “private,” “managed,” or “public.” Variants that are not publicly shared are marked “private” (only visible to the depositing center) or “managed” (shared with one or more consortia) and will not be visible for users who are not part of the same project or consortium, respectively.

## Search and Browsing

Over the years, registered and nonregistered users have greatly benefited from the powerful built‐in search engine of DECIPHER. One of the most significant recent developments pertaining to this search functionality is the implementation of an advanced search mechanism. Specifically, users can either conduct basic queries using simple terms (search by gene, position, and phenotype, among others) or tailor their search results by combining terms from two or more fields (see Fig. 2B). A search on one phenotype term from the HPO will also search for matches with all its descendant HPO terms. To speed up the search interaction, a newly introduced autosuggestion feature enables users to browse and select an option from an automatically retrieved list of known values that match their input. The values are instantly updated while typing, and are grouped into predefined categories according to their origin (Fig. 2B). The outcome of each search is displayed in a tabular format, accompanied with interactive visual representations. In addition to search, DECIPHER also offers a means of interactively browsing regions of the genome with accessible patient data and reference data by using gene symbols, chromosomal bands, or positions. This enables identification of variants that overlap the query term for further analysis.

## Data Visualization

As a means of enhancing interpretability, DECIPHER offers a plethora of modern purpose‐built data visualization aids that range from static graphs to interactive web tools such as the Genoverse genome browser (http://genoverse.org). Genoverse is a powerful, fully customizable, back‐end‐independent genome browser that allows users to interactively explore genomic data. In DECIPHER, Genoverse is configured to display tracks containing copy‐number and sequence variation data against various pathogenic (Human Gene Mutation Database [HGMD] [Stenson et al., 2009], International Standards for Cytogenomic Arrays (ISCA), etc.) and population resources (dbSNP, common CNV, etc.). Additionally, a number of tracks include trusted variants from Gene Reviews [Pagon, 2006], Morbid genes [Amberger et al., 2015], and DDD research variants [Deciphering Developmental Disorders Study, 2015], among others have recently been added.

In addition to the standard features one would expect from a modern browser, Genoverse offers additional functionality including “focus here” for quick zoom‐in (focus) on a specific feature of interest, in‐track filters, and track duplication to apply and view different selection or visualization criteria to data. For instance, a gene track may be duplicated to show protein‐coding genes in one, noncoding genes in another, or colored by their haploinsufficiency scores in one, and only known developmental disorder genes in the other. Finally, Genoverse saves the configuration of the user's tracks (order, visibility, filters, and properties) to customize display of information most relevant to them.

Genoverse employs the latest web technologies that offer dynamic “on‐the‐fly” data visualization, customizable views, interactive scroll and zoom functionality, and support for various data formats and sources. The robust design of Genoverse is amenable to straightforward incorporation in any independent web interface and scientific project. Within DECIPHER, Genoverse constitutes the basis of several advanced visual aids such as the fully interactive karyotype representation, which assists with the identification of common patterns of variation. As DECIPHER now contains many thousands of variants, we have implemented a histogram view of the variant count on the karyotype (Fig. 2C). This provides an elegant and intuitive way of assessing the spread of variation across the genome and allows a user to quickly identify regions of interest to their query. The users can interact with each position in the histograms, and with chromosome bands to acquire a closer view of the individual features at that specific position.

## Finding Similar Patients

DECIPHER has pioneered novel mechanisms for the identification of patients with similar genotypes. As a means of highlighting similarities and patterns in the external developmental characteristics between deposited and overlapping patients, all common phenotypes are highlighted. Detailed information about shared genes within deletions or duplications may highlight genes of particular interest for deeper investigation. Pie charts allow easy visualization of the spectrum of overlapping patients based on different criteria such as inheritance and pathogenicity, among others. Recently, the identification of CNVs with similar functional impact has been enhanced to include CNVs that affect a common gene but without a positional overlap. Each overlap is scored according to its functional similarity, which aids in prioritizing the variants that present the most functionally similar genotypes (Fig. 2D).

## Syndromes and Gene Disorders

DECIPHER maintains up‐to‐date annotations of reference data that are collated from different sources and include current and previous gene symbols, gene chromosomal locations, and cross‐reference identifiers to other resources that include sequence databases, protein domains, interactions, and protein structure databases. This permits the display of various properties associated with genes that are affected by a variant deposited in the database. Known pathogenic copy‐number and sequence variation data are collated from trusted resources such as the International Standards for Cytogenomic Arrays (ISCA) [Riggs et al., 2012], ClinVar [Landrum et al., 2014], HGMD public [Stenson et al., 2009], and the Leiden Open Variation Database (LOVD) locus‐specific data [Fokkema et al., 2011] for assisting variant interpretation. In addition, DECIPHER has also collated over 3,500 variants known to be associated with over 300 gene disorders from GeneReviews [Pagon, 2006] to aid interpretation of deposited SNPs. This information can be directly accessed from the gene disorder pages (https://decipher.sanger.ac.uk/disorders#diseases) or seen on the interactive Genoverse genome browser from patient genotype pages and from the main DECIPHER Website.

DECIPHER syndromes (https://decipher.sanger.ac.uk/disorders#syndromes) are a curated collection of well‐known syndromes caused by deletions and duplications. Individual syndrome pages present expert‐reviewed clinical synopses, size, and nature of deletion or duplication, a list of genes contained within the aberration, literature references, and links to appropriate support groups. Syndrome information is also presented on patient pages, in the Genoverse genome browser and via search results, thereby ensuring that it is readily available to aid interpretation.

## Research Data

The DECIPHER platform also allows for high‐quality research data to be shared globally. Some data in DECIPHER have been populated from published research to help identify and interpret a deposited variant, and large research projects such as the DDD project [Wright et al., 2014] have also made available many plausibly pathogenic variants of unknown significance in genes previously unlinked to genetic disorders (https://decipher.sanger.ac.uk/ddd#research‐variants/snvs). These variants include validated functional de novo variants and rare loss‐of‐function homozygous, compound heterozygous, and hemizygous variants in genes lacking both an OMIM Morbid status and common loss‐of‐function variants. The aim here is to help bring together geographically distinct clinical and research groups that may have patients or data on these genes to maximize the identification of new disease genes in as‐yet undiagnosed individuals. In the first 5 months alone, the publication of these variants has resulted in more than 40 international collaborative links being forged between clinicians with similar genotypic presentation.

## Matchmaking in DECIPHER

**Figure 3:**
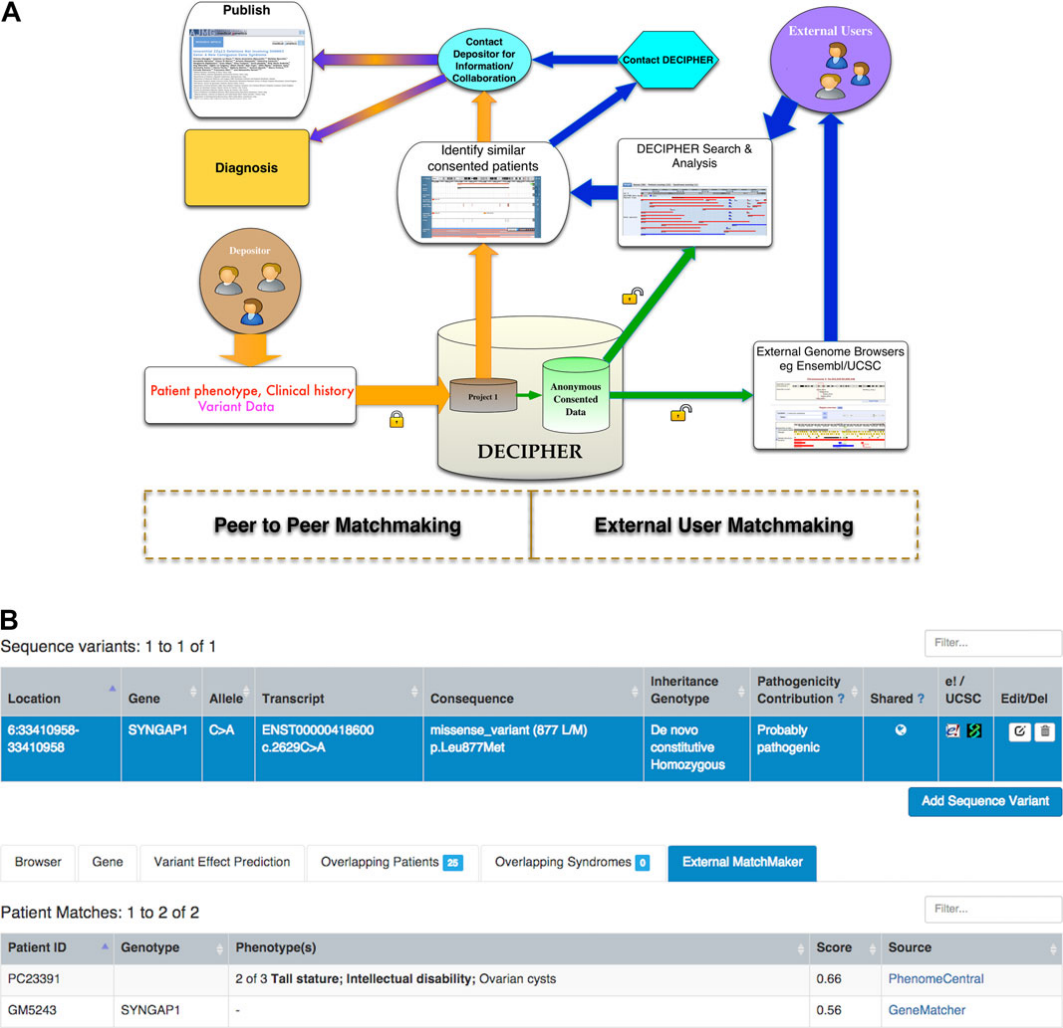
Facilitating matchmaking in DECIPHER. **A**: Registered depositors of the DECIPHER platform can deposit their patient data into their center and then find other anonymized patient‐consented data similar to their patient in the database and make direct contact with other depositors. Anonymous patient‐consented data are also shared with Ensembl and UCSC genome browsers. External users can find patient‐consented data via search or Ensembl and UCSC genome browsers and initiate contact with a depositor for information or collaboration facilitated by DECIPHER. **B**: A mock‐up of the implementation of the MatchMaker Exchange (MME) application programming interface (API) in DECIPHER. Depositors who share their anonymous data will benefit from finding other patients in other databases that are part of the MME API project.

The DECIPHER project has been highly successful in providing a platform to bring together clinicians and researchers from different parts of the world through sharing anonymized patient genotype and phenotype data, leading to collaborations, exchange of information, publications (Fig. 1C), and diagnoses. A schematic of how matchmaking works in DECIPHER is shown in Figure 3A. DECIPHER facilitates matchmaking via many routes including:

- Internal matchmaking: registered DECIPHER depositors can deposit patient data via the online Web interface, and use its built‐in functionality to detect patients with similar genetic and phenotypic features. Once a similarity (match) has been identified, the DECIPHER platform enables users to get directly in touch with the depositing clinicians from the list of matching patients using its in‐built contact form.
- External matchmaking: external users can exploit the search and browsing functionality, and query through publicly available anonymized patient data in order to discover potential matches and initiate contact with the depositor via DECIPHER. This provides a means of bringing depositors and clinicians into contact, leading to successful collaboration and exchange of information.
- Anonymized, patient‐consented data deposited in DECIPHER data are also shared with third‐party software variant analysis vendors and the popular UCSC and Ensembl genome browsers, thereby maximizing potential reach of these data for the purposes of finding similar matches elsewhere.
- Bulk data: DECIPHER makes all consented, anonymized data available for methods development and meta‐analysis [Huang et al., 2010; Johansson and Feuk, 2011; Boulding and Webber, 2012) as an encrypted downloadable bulk data file for bona fide academic researchers under a formal Data Access Agreement. A separate Data Display Agreement is required for visualization purposes. Both agreements can be found on the DECIPHER website.

The international MatchMaker Exchange (MME) project (http://matchmakerexchange.org), of which DECIPHER is a founding member, is a new, collaborative, worldwide initiative aiming to identify genetic causes in patients with rare disorders based on the automated exchange (matchmaking) of genetic and phenotypic information between different databases housing genotype and/or phenotype data on rare disease patients, via an application programming interface (API). Each team within the MME project is in charge of constructing its own unique federated exchange platform used to aggregate and share instances of pathogenic variation among multiple independent research and clinical sources, which commonly conduct analysis in isolation.

DECIPHER has implemented version 1.0 of the MME API as a means of matching its publicly available anonymized patients with external cases of similar genotypes and phenotypes. A user interface (under development) will allow a DECIPHER depositor to find potential matches in other databases that implement the MME API (Fig. 3B). We are confident that external users from other collaborating databases will greatly benefit from the extremely rich set of patient‐consented phenotypic data that DECIPHER has to offer in return. In the cases of a successful match, an external user can submit a request to contact the clinicians of consented patients within DECIPHER. Applications that utilize DECIPHER's implementation of the MME API will be able to provide their users with a link to the corresponding DECIPHER patient and rank patient similarity using a matching score returned by DECIPHER. This score is calculated based on the work of Guo et al. (2006), and will be in the range between 0 and 1, where 0 represents no match and 1 a perfect match. DECIPHER's contact form can then be used by that submitter to send a contact request to the DECIPHER team, who will then facilitate communication with the depositing center.

## Summary

Since its inception in 2004, DECIPHER has played a pivotal role in aiding clinical diagnosis of patients with developmental disorders by encouraging sharing of anonymized patient consented phenotype and genotype data and providing a platform for contact and collaboration when patients sharing similar features are identified. DECIPHER has grown significantly in the last 5 years, going from ∼2,000 consented patient records in 2009 to over 17,000 in 2015. These additional shared data provide a greater scope (roughly 50‐fold) for matchmaking and are in turn reflected by the increase in the number of international collaborations and subsequent publications in peer‐reviewed scientific literature. By expanding DECIPHER's remit from copy‐number data to include sequence‐level variation, we have provided comprehensive coverage of genomic space and kept pace with advances in genetic technologies. In 2014, DECIPHER was completely redesigned in order to update the service to take advantage of better web security and technologies, and to be able to offer enhanced tools and functionality in the future. We are working toward further enhancing DECIPHER's collaborative matchmaking effort by providing new notification mechanisms (matchmaking alerts) and better improved algorithms (genotype and phenotype scoring). Work is also underway to develop tools to facilitate seamless deposition of anonymized patient data from third‐party analysis software. Finally, we are looking to improve user interactions by providing simpler deposition interfaces, better search methods, improved visualization, and additional statistical tools for interpretation of deposited data.

## Contacting DECIPHER

The DECIPHER platform is under continual development and we are open to all ideas and suggestions for improving the user interface or incorporation of tools for variant interpretation. We have a dedicated helpdesk email address, decipher@sanger.ac.uk, for feedback, bug reports, or any other query. A mailing list for DECIPHER news and notifications is also available (http://publists.sanger.ac.uk/mailman/listinfo/decipher‐announce).
